# Satellite-Based Assessment of Grassland Conversion and Related Fire Disturbance in the Kenai Peninsula, Alaska

**DOI:** 10.3390/rs11030283

**Published:** 2019-02-01

**Authors:** Katherine A. Hess, Cheila Cullen, Jeanette Cobian-Iñiguez, Victor Lenske, Jacob S. Ramthun, Dawn R. Magness, John D. Bolten, Adrianna C. Foster, Joseph Spruce

**Affiliations:** 1 NASA DEVELOP National Program, NASA Langley Research Center MS 307, Hampton, VA 23681, USA; 2 Department of Geographical Sciences, University of Maryland, College Park, MD 20742, USA; 3 NOAA-Crest Center, The City University of New York, Bronx, New York 10453, USA; 4 Department of Mechanical Engineering, University of California, Riverside; Riverside, CA 92521, USA; 5 Science Systems and Applications, Inc., 10210 Greenbelt Rd, Lanham, MD 20706, USA; 6 Department of Geography, University of South Carolina, Columbia, SC 29208, USA; 7 Kenai National Wildlife Refuge, U.S. Fish and Wildlife Service, Soldotna, AK 99669, USA; 8 Hydrological Sciences Laboratory, NASA Goddard Space Flight Center, Mail Code 617.0, Greenbelt, MD 20771, USA; 9 Universities Space Research Association, 7178 Columbia Gateway Dr, Columbia, MD 21046, USA; 10 NASA Stennis Space Center, Stennis Space Center, MS, United States

**Keywords:** wildfire, grassland, risk modeling, land cover, change detection, Alaska

## Abstract

Spruce beetle-induced (Dendroctonus rufipennis (Kirby)) mortality on the Kenai Peninsula has heightened local wildfire risk as canopy loss facilitates the conversion from bare to fire-prone grassland. We collected images from NASA satellite-based Earth observations to visualize land cover succession at roughly five-year intervals following a severe, mid-1990’s beetle infestation to the present. We classified these data by vegetation cover type to quantify grassland encroachment patterns over time. Raster band math provided a change detection analysis on the land cover classifications. Results indicate the highest wildfire risk is linked to herbaceous and black spruce land cover types, The resulting land cover change image will give the Kenai National Wildlife Refuge (KENWR) ecologists a better understanding of where forests have converted to grassland since the 1990s. These classifications provided a foundation for us to integrate digital elevation models (DEMs), temperature, and historical fire data into a model using Python for assessing and mapping changes in wildfire risk. Spatial representations of this risk will contribute to a better understanding of ecological trajectories of beetle-affected landscapes, thereby informing management decisions at KENWR.

## 1. Introduction

In the mid-1990’s, North America’s largest recorded outbreak of spruce beetles (*Dendroctonus rufipennis* Kirby) killed nearly 5 million acres of forest on and around south-central Alaska’s Kenai Peninsula [1]. Stands of White and Lutz spruce (*Picea glauca*; *Picea x lutzii*) were particularly vulnerable, with the boreal forest ecosystem on the peninsula’s western lowlands suffering mature tree mortalities as high as 87% in some areas [2]. Kenai’s boreal spruce stands typically exhibit high tree densities constituted by specimens between 15-30 m in height and up to 60-90 cm diameter at breast height (DBH) [3], making these forests both attractive and particularly susceptible to the rapid contagion of parasitoid species. Spruce beetle-induced mortality results in foliar desiccation (“red phase”) before an eventual needledrop (“gray phase”), thus opening the canopy and often permitting ecological succession towards more grass-dominant ecotones [4]. Such infestations, in addition to causing habitat loss, also harm the timber economy, detract from regional tourism, increase risk of property damage due to treefall, and cause potential shifts in the fire regime [5] (pp. 195-197). Beetle-specific mortality has been observed to desiccate and weaken trees, leading to greater heat output and likelihood of collapse that puts firefighters at greater risk of injury [4]. Both beetle outbreaks and fires are of serious concern to wildlife managers in the region. Because the Kenai Peninsula is one of Alaska’s most densely populated boroughs and is a cornerstone of the state’s tourism economy, the ability to forecast and mitigate these disturbances is of high value to a variety of local stakeholders.

Southern Alaska has been witnessing another rapid surge in spruce beetle populations since 2014 [1], and local climatic conditions are becoming increasingly favorable for both spruce beetles and wildfire. The peninsula’s vegetation cover has transitioned to dry biome as the region has experienced a 1-2 °C temperature increase over the last half-century [6]. Previous studies have not only suggested that increasing temperatures correlate with fire risk, but also that multi-year spans of above-average summer temperatures may positively correlate with risk of beetle infestation [2,7]. Warmer, drier conditions increase the rate at which spruce beetles reach maturity, remove climatic barriers to the spread of infestations, and weaken spruce trees’ natural defenses against parasitoid beetles, such as the production of resin [8] (pp. 604-606). The beetle outbreak of the mid-1990’s notably differed from previous infestations in the region. Specifically, it was not preceded by a clearly identifiable and succinct disturbance, such as a fire or windfall, which would have jeopardized the defense mechanisms of spruce populations [7] (p. 220). Previous studies in both Alaska and the continental United States have suggested that droughts or consecutive years of above-average temperatures may be enough to make spruce forests vulnerable to regional beetle outbreaks [7,9]. However, these studies were based on a relatively small sample of recorded outbreaks.

Although studies of the continental United States [10,11] have concluded that wildfire frequency and size are not significantly increased by beetle-induced damages, others suggest that the opposite may be true in Alaskan boreal forests where canopy loss often lends itself to grassland conversion [12,13]. Increased grass cover, paired with the accumulation of dried foliage after beetle outbreaks, creates conditions conducive to the surface fuel ignition typical of boreal forests [13]. A shifting fire regime is of particular concern in Kenai, as the peninsula’s biome is characterized by fire return intervals (FRIs) of approximately 400-600 years [12]. Even where grass encroachment is high, Kenai’s spruce forests have historically exhibited sufficient density to quickly restock saplings after beetle outbreaks [14]. In recent years, Kenai National Wildlife Refuge (KENWR) ecologists have observed this rate to have sharply decreased. It is unclear how the compounding threats of rising temperatures, increased wildfire frequency, and beetle outbreaks will shape long-term ecological succession on the Kenai Peninsula. However, we hypothesize that areas that have converted to grassland subsequent to spruce beetle-induced forest mortality, as well as forest adjacent to grassland ecotones, will exhibit greater risk of wildfire, unlike patterns observed in studies of the continental United States.

### 1.2 Study Area

**Figure 1.**
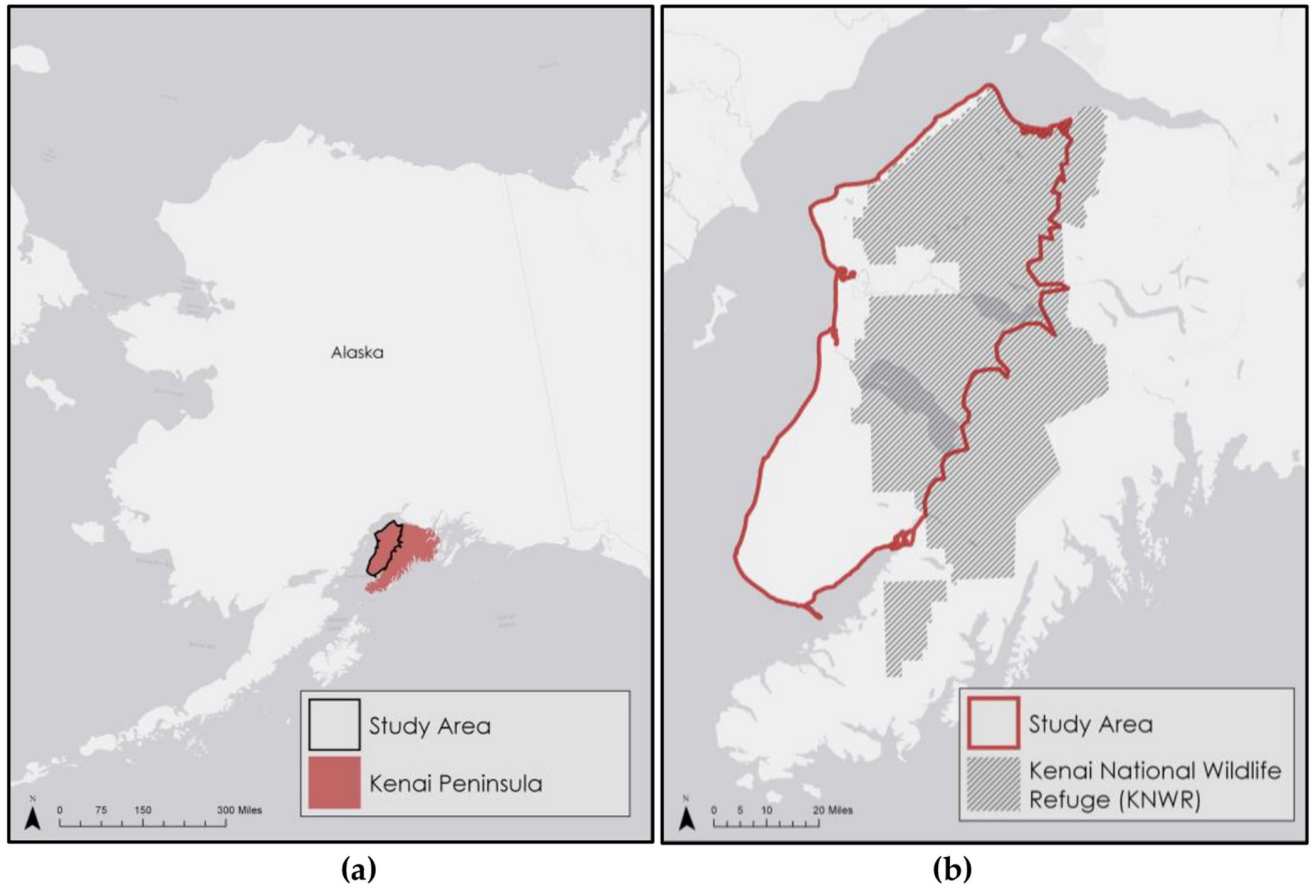
Location of the study area and the Kenai National Wildlife Refuge within Alaska.

The Kenai Peninsula covers an area of roughly 26,700 km^2^ and is located between 59° 8′ and 61° 4′ N latitudes and 152° 2′ and 147° 55′ W longitudes in south-central Alaska (Figure 1a). Containing the Kenai Mountains along its eastern half, the peninsula ranges in elevation from sea level to around 2,100 m. Our study area (Figure 1b) was situated on the flatter interior coast along the Cook Inlet (59° 35′ to 61° 3′ N latitude, 149° 58′ to 151° 53′ W longitude). These spruce forest lowlands contain most of the peninsula’s urban areas and have witnessed most of the peninsula’s recorded fires. The decision to exclude the Kenai Mountains was intended to streamline our classification process by minimizing non-vegetated land cover. The study area is divided roughly in half by Tustumena Lake. The northern half contains most of the KENWR and consists largely of terrain that is low-lying, marshy, and characterized by the dominance of the more beetle-resistant black spruce (*Picea mariana*). The southern half, including the Caribou Hills region, is more topographically varied and dominated by white and Lutz spruces (*Picea glauca*; *Picea x lutzii*) which are particularly vulnerable to beetle damage [3].

### 1.3 Objectives

The aim of our research was to explore the potential utility of satellite imagery in characterizing the relationship, if one exists, between grassland conversion and emergent wildfire risk in Alaskan boreal forests. The objectives comprising this process were to 1) build an optimized land cover classification system tailored to detecting grassland conversion in this particular biome, 2. use this system to map grassland conversion and detect land cover changes from 1995 (at the apex of the 1990’s beetle outbreak) to the present, and 3) develop a model for quantifying and mapping emergent wildfire risk resulting from this conversion. The KENWR, administered by the U.S. Fish and Wildlife Service (USFWS), provided *in situ* data and consultation for this research with the intent of better understanding ecological trajectories in a shifting disturbance regime. Our research will support the KENWR’s decision-making process to improve planning for fire control and ecosystem restoration efforts. This research will help land managers better predict changes in forest structure and fire regime, not only protecting adjacent stakeholders in Kenai from the socioeconomic damages of forest loss, but also yielding lessons that can be transferable to both Interior Alaska and Canada’s Yukon Territory where large expanses of similar spruce-dominated boreal forest are present.

## 2. Materials and Methods

### 2.1.1 Data Acquisition - Classification & Vegetation Transition

We used the United States Geological Survey’s EarthExplorer data portal (https://earthexplorer.usgs.gov/) to access NASA’s Landsat Collection 1 Level-2 (on demand) atmospherically corrected surface reflectance imagery (Path 069, Rows 017-019). A threshold of 20% for land cloud cover was used to define significant cloud interference. Scenes surpassing this value were queried out to minimize the amount of data lost due to eventual pixel masking. The included imagery dates were selected to constitute a complete, yet concise, timeline of Kenai’s ecological succession in the wake of the 1990s spruce beetle infestation. Landsat 5 Thematic Mapper (TM), Landsat 7 Enhanced Thematic Mapper Plus (ETM+), and Landsat 8 Operational Land Imager (OLI) scenes captured grassland conversion from the apex of the 1990’s infestation to the present. As a means of eventual validation for our land cover classification process, we accessed supplemental high-resolution (30 cm) imagery from DigitalGlobe’s WorldView-3 (WV-3) satellite sensor, via the NextView Licensing agreement with the National Geospatial-Intelligence Agency.

Partners at the KENWR provided ancillary vector data that include fire history data from 1989 to the present and a series of points delineating 2015 land cover ground-truthed from sample plots. KENWR ecologists also provided a draft of a 2017 vegetation type classification of the peninsula that is currently being validated on the ground. In addition to the WV-3 imagery, these layers informed the development and validation of our land cover classification system and served as a reference for our wildfire risk model.

### 2.1.2 Data Acquisition - Fire Risk Analysis

Our fire behavior analysis scheme may be considered to fall under the categories of fire risk modeling and GIS-derived data driven modeling. Generally, fire behavior is modeled across a wide array of temporal and spatial scales. At the temporal scale, short term modeling encompasses real-time and daily predictions required for activities such as fire suppression, while long term modeling involves assessment of static fire risk factors and is thus it is applicable for seasonal or longer term activities such as fire planning [15]. At the spatial scale, fire behavior can be examined from the micro scale assessing kinematic fire behavior to the very large scale encompassing global fire trends. In this work, we assessed the impact of decade-long vegetation change on fire risk. Therefore, we capture the changing fire ecology at this time scale through a long term modeling approach. Thus, under these considerations, our modeling approach can be classified as long-term regional modeling of fire behavior.

In deriving our fire risk model, we followed existing methodologies to generate a model that integrates parameters within the weather, topography, and fuel categories of fire drivers [16–20]. Historical fires obtained from the Alaska FIREHouse database prepared by the Alaska Fire Service for 2001, 2008, 2010, 2012 and 2014 were selected as representative fire-events for algorithm training. A 10m resolution Digital Elevation Model (DEM) from the United States Geological Survey’s (USGS) 3D Elevation Program (3DEP) was used to produce elevation, slope, and aspect information. Classifications of land cover type derived from Landsat imagery, as explained in section 2.2.1, were used as the vegetation component, and monthly average temperature data was acquired from the National Scenarios Network for Alaska and Arctic Planning (SNAP). As a comparison for both our classification and fire risk systems, we incorporated data from the USGS LANDFIRE Reference Database (LFRDB), containing classifications of vegetation type, structure, disturbance patterns, and fire regime.

### 2.2.1 Data Processing - Classification

Landsat spectral bands for three adjacent scenes were merged into a composite mosaic before being clipped down to remove the peninsula’s Eastern mountains. To expedite the process of land cover classification, we created a mask from the pixel Quality Assessment (‘pixel_QA’) bands in the Landsat download package to exclude cloud cover, cloud shadows, snow/ice, and significant water bodies. The removal of these pixels reduced raster processing times and streamlined classification by minimizing the interference of unwanted spectral signatures. In addition to the original Landsat bands, we also calculated the normalized difference vegetation index (NDVI) to better differentiate between grassland and forest cover. This additional layer, derived from the original bands, supplemented the distinction of different vegetation types by reflecting foliar productivity.

**Figure 2.**
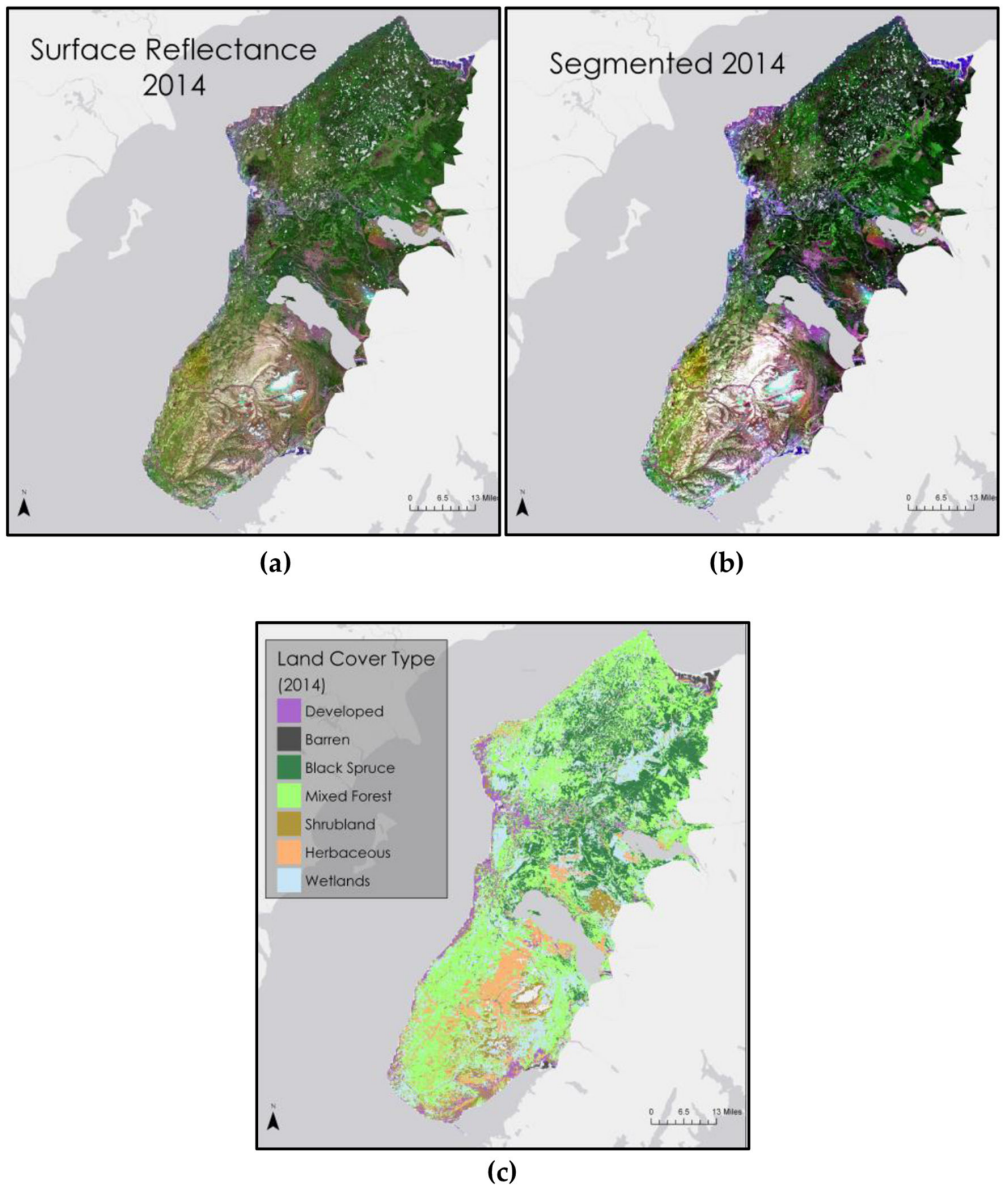
Land cover classification process for 2014 imagery.

We stacked the original surface reflectance bands (Figure 2a) with the NDVI band, and then used unsupervised segmentation to group neighboring pixels with similar spectral signatures into objects for easier classification (Figure 2b). Utilizing the high resolution WV-3 imagery, 2015 ground-truthed land cover points and the draft vegetation type map from ecologists at the KENWR, we then created a set of training samples for the 2014 image. The training samples, along with the segmentation image and the stacked 2014 surface reflectance and NDVI bands, were put through a vector-supervised machine classification. The resulting image (Figure 2c) shows the locations of developed, barren, black spruce forest, mixed forest, shrubland, herbaceous, and wetland land cover.

### 2.2.2 Data Processing - Vegetation Transition

**Figure 3.**
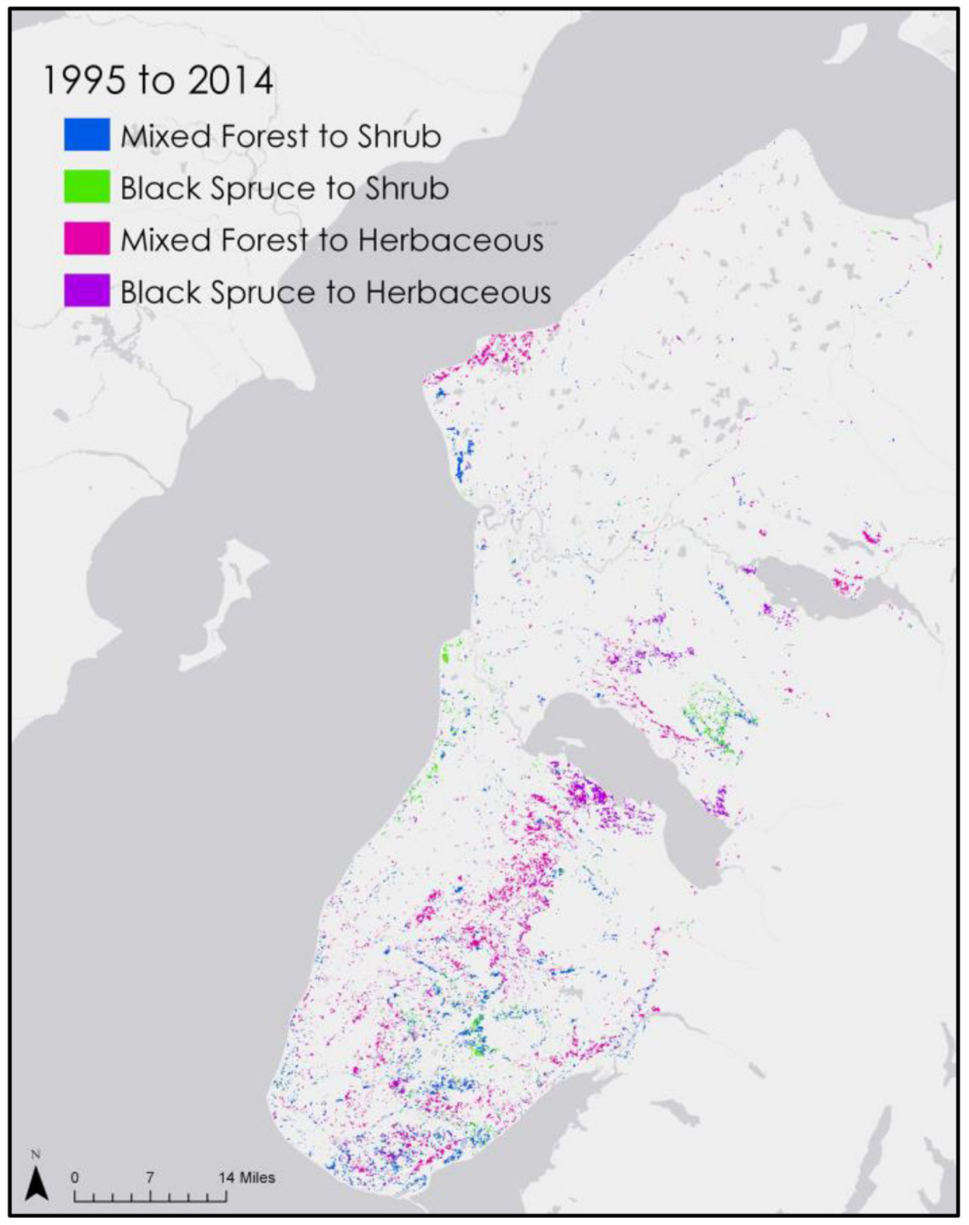
Conversion from forest to herbaceous/ shrubland based on land cover classifications from 1995 and 2014.

The vegetation transition between 1995 and 2014 was computed using two measurements: land cover classification and NDVI. For the classification transition detection, we used classified images from 1995 and 2014 that we created using the methodology in section 2.2.1. Each pixel in these images has a value between 0-6 based on their classified land cover type (0- Developed, 1- Barren, 2- Black Spruce, 3- Mixed Forest, 4- Shrubland, 5- Herbaceous, 6- Wetland). The 2014 values were multiplied by 100 and the 1995 values were subsequently subtracted. We took the resulting image and symbolized it with unique values, then kept only the values 397 (which indicates a pixel that has transitioned from mixed forest to shrubland), 398 (black spruce forest to shrubland), 497 (mixed forest to herbaceous), and 498 (black spruce forest to herbaceous). The resulting map shows pixels that have transitioned from forest to grassland (Figure 3).

**Figure 4.**
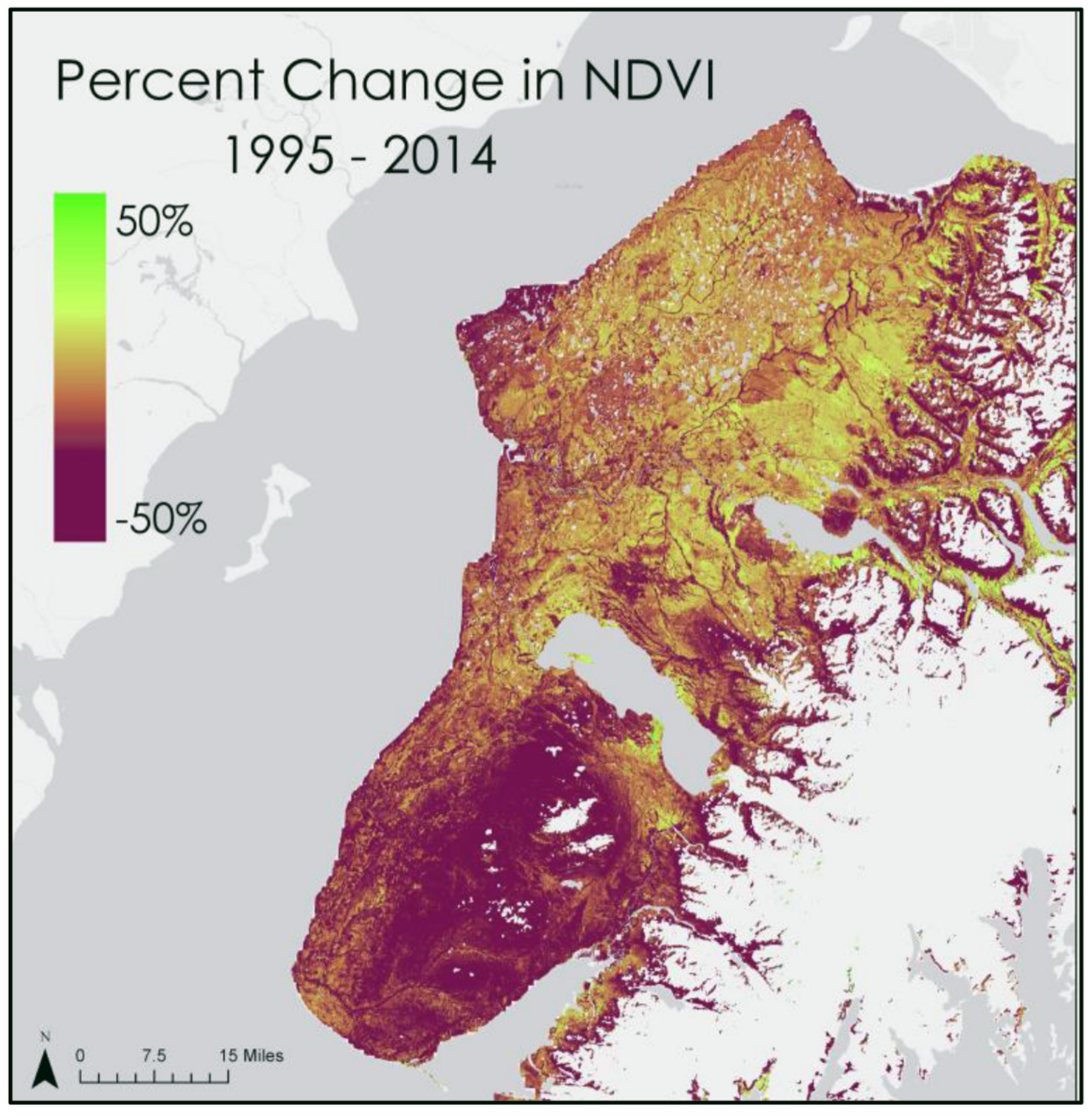
NDVI change between 1995 and 2014.

NDVI change with respect to time was calculated using the following equation (1). The resulting map (Figure 4) showed the percent change in NDVI from 1995 to 2014.

(1) $${\mathrm{NDVI}}_{\text{Change}}=\frac{{\mathrm{NDVI}}_{\text{Current}}-{\mathrm{NDVI}}_{\text{Historic}}}{{\mathrm{NDVI}}_{\text{Historic}}}$$

### 2.2.3 Data Processing - Fire Risk Model

A logistic regression was applied to calculate the probability of the analysis outcome being a fire-event or a non-fire-event. An algorithm of this kind requires sample data from actual fire-event and non-fire-event conditions so it can learn to distinguish between the two. Hence, random non-fire points were generated for each corresponding fire season. To overcome spatial heterogeneity and correlation associated with a large study area, random points creation is limited to each vegetation class. For a given number of actual fire-events in one vegetation class, matching numbers of random points are created within the extent of that class. This approach reduces the area to that of the class extent, and also assures that random points are created with at least 10 meters of distance from the actual event and from each other. Table 1 shows the fire and non-fire events used to train the algorithm.

The final input dataset was divided using the Hold-Out method, in which data is divided into a “training” and a “validation” set. In this work, fire-events and non fire-events were divided into approximately 70% training and 30% validation data. Once all data is defined, a Python subroutine is used to calculate the Z factor coefficients for the logistic equation. The algorithm is then translated into a band math equation where the final risk map for the area is configured.

## 3. Results

### 3.1. Vegetation Transition

The classification transition assessment focused on pixels that were classified as mixed or black spruce forest in 1995, and were then classified as shrubland or herbaceous in 2014. The resulting map (Figure 3) shows the change is clustered in the Southern area of the peninsula where the worst of the spruce beetle damage occurred around 1995-96.

Furthermore, it was found that NDVI change between 1995 and 2016 aligned with the results from the classification change detection, showing a high percentage of change south of Tustumena Lake (Figure 4).

### 3.2. Fire Risk Model

The logistic equation, indicated in equation (1), estimates the coefficients related to each factor representing the change in “log-odds” with a binary variable.

(2) $$\text{Probability}(\text{Fire}=1)=\frac{1}{1+e^{-Z}}$$

The coefficients are estimated via the Maximum Likelihood Estimate (MLE) method, which identifies the coefficients that make the log of likelihood as large as possible or as small as possible. Therefore the Z factor for the logistic regression of the fire risk model becomes:

(3) Z=−((Aspectx−0.0117)+(Slopex0.6532)+(Elevationx0.0011)+(Temperaturex−0.1626)+(Vegetationx0.2177)+3.7711)

In equation (2), P tends to 1, as Z in equation (3) increases. Mathematically, the probability of a fire-event tends towards 1 (fire), as Z increases, and towards 0 (no-fire) as Z decreases. Hence any variable directly proportional to a fire-event has a positive coefficient in equation (2) and vice versa.

Vegetation in this case is a categorical variable; this means that numerical values are assigned to represent each class. Increasing values are assigned based on the number of actual historical fires in each category. For example, in a dummy scale ranging from 1-7, Barren class is assigned a 1 and mixed forest is assigned a 7. Table 2 shows each variable corresponding coefficient and its corresponding significance.

**Figure 5.**
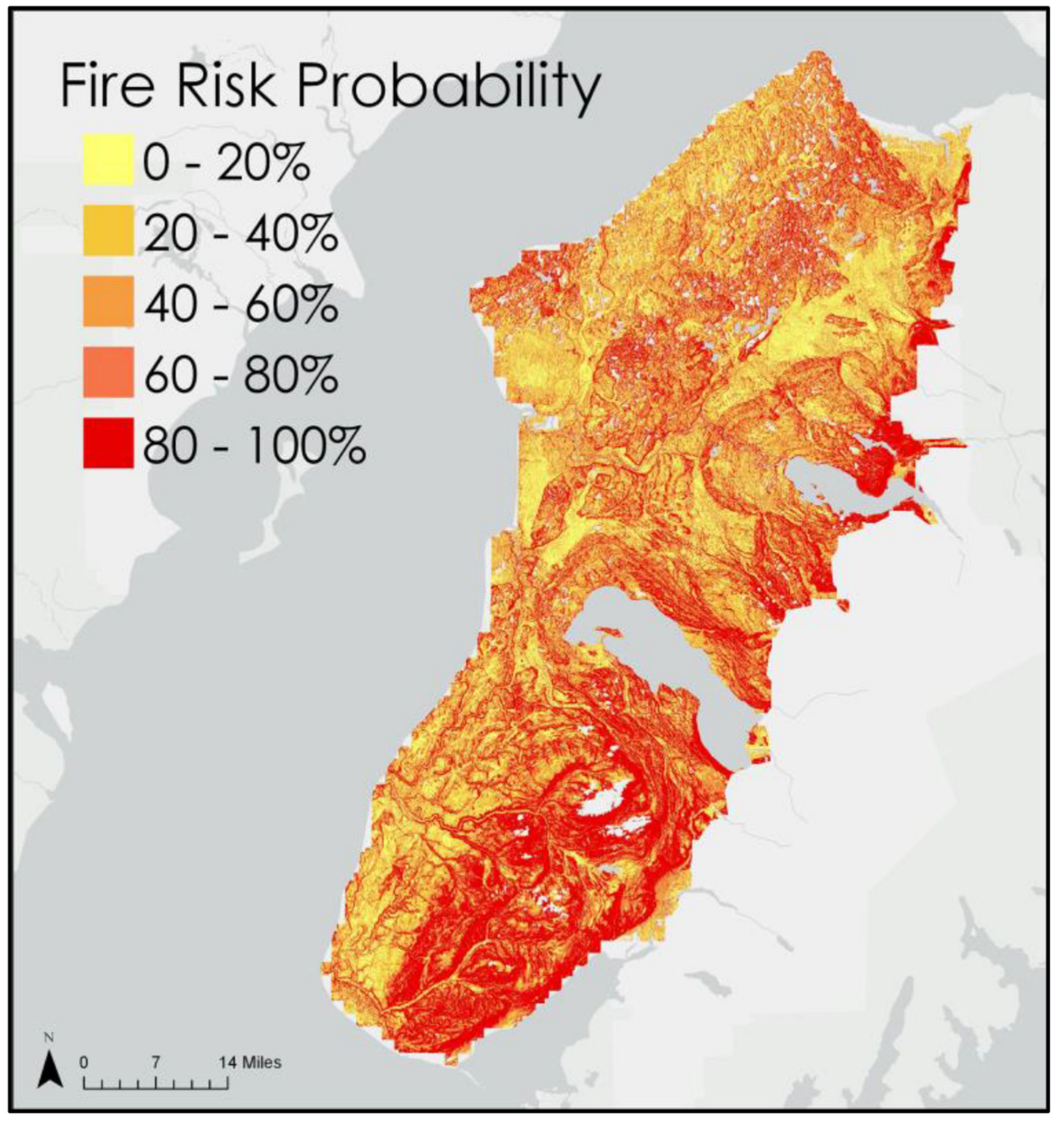
Fire Risk Map

Using the ArcGIS Pro raster calculator, all raster variables were multiplied by their appropriate weight and plugged into the logistic regression equation, resulting in the fire risk probability map shown in Figure 5.

As described above, the Hold-Out method was used for validation. The data was divided randomly into a 70% - 30% ratio for subsets as “model training” and “model validation” respectively. Validation results show that the model predicts cases correctly at a 90% accuracy.

**Figure 6.**
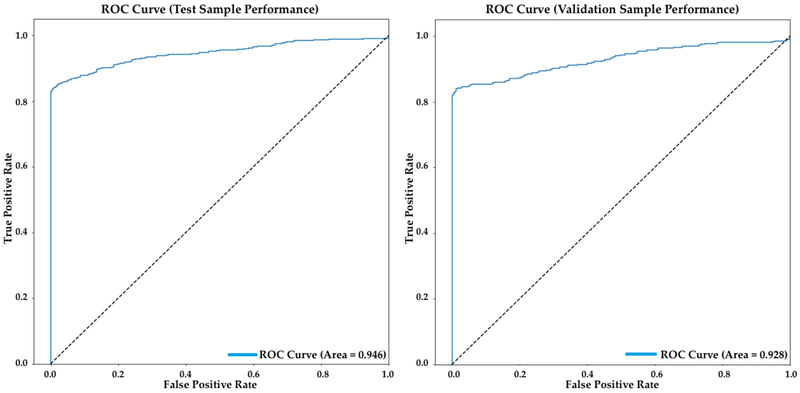
AUC 0.5 cut-off value

The AUC (area under the curve) is a measure of how the model performs by presenting the trade-off between true and false positive proportions measuring the accuracy of the analysis. An area of 1 represents a perfect test, while an area of 0.5 is considered a failed model. The resulting value for the AUC at a 0.5 cut-off value of the training set is 0.944 and 0.928 for the validation set, hence, the 0.5 cut-off value is the selected threshold for the fire and non-fire decision. Figure 6 below shows the AUC curve for the datasets.

## 4. Discussion

### 4.1 Spruce Beetle Impact

Our change detection analyses of NDVI composites and land cover classifications corroborate KENWR landscape ecologists’ observations that spruce beetle-induced mortality of forest is conducive to the establishment of grassland. Areas that were severely impacted by the spruce beetle infestation of the 1990s, such as the Caribou Hills region south of Tustumena Lake, exhibited widespread conversion of forest cover to either herbaceous or shrubland land cover types between images classified in 1995 and 2014. These areas generally exhibited greater wildfire risk in our model.

### 4.2 Fire Risk Model

The model resulted in a negative coefficient for our temperature variable. In a logistic regression setting, a negative coefficient represents that the factor in question is directly inversely proportional to the likelihood of fire. This may be due to a couple of reasons. First, as stated by other authors [21–23] some variables can be proxies of each other. Slope for example, is a proxy measure of altitude, which at the same time, regulates temperature and hence vegetation. Second, the temporal resolution of surface temperature used to build this model was based on monthly averages due to its availability. This average temperature may not be the adequate value as the monthly average temperature may smooth the real temperature value on the day of the fire. Hence, it is recommended that for future analysis, temperature be considered on a daily basis or be excluded from the model.

### 4.2 Limitations

The size of our study area and limited access to ground-truthed sample data necessitated a dependence on existing literature to justify the inclusion and weighting of individual input variables for our algorithm. Under ideal circumstances, *in situ* validations of the data would be used maximize our confidence levels in the relationships between each respective input variable and the resulting risk of wildfire. Varying availability of certain climatic data inhibited our capacity to address dynamic parameters. Acknowledging the likely benefit that soil moisture data would contribute to the algorithm, it was not factored into our research due to the lack of a single continuous data source across the span of our study period. A relative dearth of consistent, ground-based measurements precluded our inclusion of certain weather parameters, such as local precipitation.

Lastly, given the wet maritime climate of the Kenai Peninsula, our selection of Landsat imagery was particularly limited by frequent, dense cloud cover. Even though the typical fire season extends from May to August, the abundance of cloud cover often precluded entire fire seasons and necessitated an analysis at multi-year intervals. This multi-year approach limited our overall number of data points and our capacity to assess environmental conditions immediately prior to certain wildfire events. Interannual phase shifts in grassland phenology were mitigated through normalization of our change detection rasters by historical values.

### 4.3 Future Directions

While our research adopts a proof-of-concept approach to modeling fire risk on the Kenai Peninsula, more advanced and comprehensive approaches for operational use would benefit from incorporating an expanded parameter set into the methodology. One such potential variable is soil moisture, which was identified by the KENWR as an important driver of fire behavior but was excluded, as stated in Section 4.2.. Incorporating soil moisture would require an assessment of the comparability of measurements between the Soil Moisture Active Passive (SMAP) satellite (launched in 2015) and its precursor satellites. Another consideration that would improve the accuracy of the fire risk model would be to account for the impact that urban environments have on the risk of ignition. Because our research was conducted in partnership with the KENWR, our area of analysis prioritized broader scopes and large tracts of wilderness, rather than the interface of wilderness and urban land cover. Including the factors of soil moisture and urban adjacency, as well as any number of additional weather variables, would greatly enhance future iterations of the model. The structure of our fire risk model is conducive to the eventual inclusion of additional, dynamic variables.

Factors that would benefit from refinement in continued research include temperature and land cover type. First, when aggregated as the monthly mean, surface temperature did not exhibit a positive correlation with the occurrence of wildfire in our study area. This is likely explained by high intra-month variability and would likely be remedied by the substitution of daily mean temperatures. Second, forest structure and vegetation type were not addressed in detail in our method of land cover classification. Given the spatial resolution of our satellite imagery and the limitations of our ground-truthed data, our classification relied heavily on generalization of vegetation types. For example, we classified the highly flammable black spruce forest as a unique land cover type, but all other tree species were combined in the mixed forest class. With additional time and resources, a greater level of detail as to the density, homogeneity, and constituent fuel species would yield valuable information about wildfire behavior and susceptibility for the region.

## 5. Conclusions

Our change detections of Landsat imagery, using NDVI and images classified by land cover type, corroborate *in situ* observations made by landscape ecologists at the Kenai National Wildlife Refuge. These findings indicate that, for the conditions considered here, spruce beetle-induced disturbance in the Kenai Peninsula’s boreal forests often results in a transition to herbaceous or shrubland ecosystems. In addressing how the change in vegetation may lead to increased fire activity, we derived a fire risk model by using a logistic regression algorithm. The algorithm was trained with historical fire data and variables representing land cover classifications, topographical parameters and temperature. This model found the highest wildfire risk to be connected to herbaceous and black spruce land cover types, aligning with the observations of the KENWR’s ecologists and existing literature.

Although previous research conducted in the contiguous United States (specifically in the Rocky Mountains) has suggested that parasitoid-induced tree mortality has only a negligible impact on emergent wildfire risk, our study has reinforced hypotheses that such patterns may not be apparent in the fire ecology of Alaska. Effects of parasitoid infestations in the Rockies were outweighed by weather variables during the burn season. Under hot, windy, and/or dry enough conditions, even healthy stands previously unscathed by beetles could burn easily. Thus, red or gray phases of parasitoid damage would have little to no difference with regards to risk. The Kenai Peninsula, however, is both a coastal environment and is situated at a much higher latitude. This results in a comparatively shorter fire season with greater precipitation and cooler mean temperatures than those found in continental case studies. As such, the disturbance regime in coastal Alaskan forests is generally typified by fires that are both less frequent (usually only occurring every few centuries) and predominantly limited to underburning (as opposed to igniting the canopy). It is plausible that beetle-induced tree mortality and emergent wildfire risk may correlate more strongly as FRIs increase. Fire ecology as a discipline would benefit greatly from further investigation into any relationship that may exist between parasitoid disturbances and fire disturbances, specifically within the context of rising temperatures and decreasing FRIs. Our study demonstrates the need for these advancements to be driven by localized case studies that take geographic and ecological nuances into account.

A better understanding of both ecological trajectories and disturbance regimes on the Kenai Peninsula carries with it great potential benefit to surrounding communities, especially in consideration of the sharp increase in local spruce beetle populations since 2014. In addition to concerns of biodiversity and habitat loss, the region is of high concern due to a relatively dense population and centrality to Alaska’s tourism industry and cultural identity. These factors, coupled with the mounting likelihood of another severe beetle infestation, create the potential for severe social and economic damages. The capacity to more accurately map ignition risk is thus widely valuable, not only contributing to a streamlined decision-making on the part of wildlife management bodies, but also potentially providing key knowledge to state and municipal governments, regional planners, the logging industry, real estate developers, and other local actors.

## Figures and Tables

**Table 1.** Vegetation class and corresponding number of fire-events and random points created

Vegetation Class	Fire-Events	Non-Fires
Developed	240	240
Barren	6	6
Black Spruce	218	218
Mixed Forest	320	320
Shrublands	104	104
Herbaceous	90	90
Wetlands	192	192

**Table 2.** Factor coefficients and their statistical significance

Factor	Coefficient	P > |z|
Aspect	−0.0117	0.000
Slope	0.6532	0.000
Elevation	0.0011	0.094
Temperature	−0.1626	0.000
Vegetation	−0.1626	0.000

**Table 3.** Model Confusion Matrix Probability of Detection (POD) and False Alarm Ratio (FAR) ^1^

	Predicted	Not Predicted	%Correct
Fire	500	74	93.4
No Fire	698	49	86.7
Percentage			90.6

1 The cut-off value is .500.

## Appendix A - Glossary

DBH: Diameter at breast height; a metric describing the dimensions of a tree
Earth observations: Satellites and sensors that collect information about the Earth’s physical, chemical, and biological systems over space and time
Ecotone: A transitional or “buffer” area between two ecosystems, such as those existing at the boundaries of forests and grasslands
ETM+: Landsat 7 Enhanced Thematic Mapper Plus
Fire regime: The type, frequency, patterns, and seasonality of wildfire as it typically occurs in a particular environment, often used to characterize different types of forest biomes
FRI: Fire Return Interval; the average number of years between significant wildfire events in a given site
Gray phase: The stage of spruce beetle-induced mortality in which the dried needles fall from the tree, accumulating as dry fuel on the forest floor, leaving the deceased trunk standing and defoliated. This is preceded by the “red phase”
KENWR: Kenai National Wildlife Refuge
LFRDB: LANDFIRE Reference Database
MLE: Maximum Likelihood Estimate
NDVI: Normalized Difference Vegetation Index. The ratio of visible red light to near-infrared light reflected from a surface, this metric is commonly used proxy for vegetation health and productivity
OLI: Landsat 8 Operational Land Imager
Parasitoid: An organism that engages in a parasitic relationship with a host organism, living attached to or within the host’s body for most of its life span before ultimately killing, sterilizing, or entirely consuming its host. This is in contrast with ‘true’ parasites, which do not necessitate the death of the host
Red phase: The stage of spruce beetle-induced tree mortality in which dry (red) needles remain on the tree; this is followed by the “gray phase”
SNAP: Scenarios Network for Alaska and Arctic Planning
Surface fire: Wildfire that spreads predominantly through a forest’s under understory vegetation. This is characteristic of fire regimes in boreal forests. This is opposed to a crown fire, which spreads between treetops
TM: Landsat 5 Thematic Mapper
Underburning: Forest fire that spreads at ground level but does not spread to the canopy
USFWS: United States Fish and Wildlife Service
USGS: United States Geological Survey
WV-3: WorldView-3 Satellite Sensor (DigitalGlobe, Inc.)
